# Adaptive Extended State Observer for the Dual Active Bridge Converters

**DOI:** 10.3390/s24082397

**Published:** 2024-04-09

**Authors:** Tan-Quoc Duong, Hoai-An Trinh, Kyoung-Kwan Ahn, Sung-Jin Choi

**Affiliations:** 1 Department of Electrical, Electronic and Computer Engineering, University of Ulsan, Ulsan 44610, Republic of Korea; 2 Department of Mechanical Engineering, University of Ulsan, Ulsan 44610, Republic of Korea

**Keywords:** extended state observer, dual active bridge converter, DC–DC converter, disturbance suppression

## Abstract

The DC–DC dual active bridge (DAB) converter has become one of the essential units for bidirectional energy distribution and connecting various renewable energy sources. When it comes to regulating the converter’s output voltage, integrating an extended state observer (ESO) offers the advantage of eliminating the need for a current sensor, thereby reducing system costs. The ESO with a high observer bandwidth tends to acquire a faster system convergence and greater tracking accuracy. However, its disturbance suppression performance will become poor compared to the ESO with a low observer bandwidth. Based on this, the adaptive ESO (AESO) is proposed in this study to make a compromise between tracking performance and disturbance suppression. When the system is subjected to a high voltage error, the observer bandwidth will increase to improve the tracking performance and decrease to enhance the disturbance suppression. In order to demonstrate that the proposed method is effective, it is compared to the ESO with a fixed observer bandwidth and the improved model-based phase-shift control (MPSC). These comparisons are made through simulation and experimental results in various operation scenarios.

## 1. Introduction

In recent years, the DC–DC dual active bridge (DAB) converter has received increasing interest due to the development of new technologies such as energy storage systems (ESSs) and electric vehicles (EVs). In comparison to other converters, the DAB converter is widely used in new energy vehicles, DC distribution networks, and DC power sources (solar photovoltaics and fuel cells), as well as the high penetration of DC loads (light-emitting diodes, computation devices, and motor drive systems) and other industrial fields. That is because of the DAB characteristics, such as the symmetrical topology, wide voltage conversion range, bidirectional power transmission, high power density, galvanic isolation, and a wide range of soft switching. Moreover, because of these benefits, the DAB converter can perform the following functions: (1) ensure that the voltage of the power sources is effectively matched to accommodate changes in various operating conditions; (2) reduce the amount of electromagnetic interference (EMI) that is coming from the outside and steer clear of the EMI that is coming from the inside that is created by the high-frequency pulse width modulation (PWM) voltage pulse; and (3) prevent the device loss that results in energy loss and the device’s heating [1,2,3,4,5,6].

There are many advanced control methods presented for controlling the DAB converter, such as linearization control [7], output current feedforward control [8], virtual direct power control [9], moving discretized control set model predictive control [10,11,12,13,14], feedforward current control [15], predictive current control [16,17], sliding mode control [18,19,20], and model-free data-driven model [21]. However, they primarily require much measurement information to control the output voltage while achieving a high dynamic performance and low computational burden. In [22], a wise combination of model-based control with the design of feedback regulators and load current feedforward regulators has a high-bandwidth control performance over the entire transmission power range without changing the controller parameters online compared to the conventional model-based control. However, it also requires information from the output current. Moreover, when many DAB converters are connected in parallel, in series, or as modules, having as many current sensors as the number of converters is necessary. This results in a significant increase in the cost of the system. Based on this, constructing an observer is a promising solution that can receive information regarding the output current of the DAB converter if the current is considered an external disturbance or observed state. The disturbance observer, the perturbation observer, the equivalent input disturbance-based estimation, and the extended state observer (ESO) are similar methods presented until this point [23]. According to [24,25], the ESO requires the smallest amount of system information. Moreover, it also allows for estimating the total disturbance, which considers internal model uncertainties, unmodeled dynamics, parasitic resistance, the tolerant drift of the inductor or capacitor, and external noises such as electromagnetic interference produced by the semiconductor switching action during operation.

However, even though the ESO is considered one of the most robust controllers with high steady-state and dynamic performance, the order of the system model is of great concern. Thus, when the system model order grows, it results in a correspondingly increased level of observer complexity. Consequently, designing the observer is a significant problem that must be overcome regarding multimodule systems. Clearly, all disadvantages mentioned above mainly come from the bandwidth of ESO.

It is important to note that the measured voltage will be affected by measurement noise, which will be amplified using a high-observer-bandwidth ESO. For this reason, the bandwidth of the ESO is typically limited to a specific value to reduce the amount of amplified measurement noise. Thus, when the conservative design is implemented, the convergence speed of the ESO is sacrificed to some degree, and the system performance in terms of disturbance suppression is reduced. The cascaded ESO was taken into consideration in [26,27,28] in order to reduce the noise that was caused by high-frequency measurements. However, the cascaded structure also makes it more difficult to tune the parameters. Moreover, the converter often operates in various scenarios, including variations in load, reference voltage, and input voltage. As a result, the controller’s performance is limited because the observer bandwidth is tuned and selected empirically at only one operation point of the converter. Several control strategies are presented to overcome the limitation of a fixed gain controller. In [29], a high-performance adaptive controller for the uncertain model of hypersonic flight vehicles has been proposed. In [30], an adaptive ESO (AESO) is used to mitigate the effects of the motor frequency variations in an interior permanent-magnet synchronous motor. There is a novel data-driven AESO-based model-free disturbance rejection control architecture for the output voltage regulation of DC–DC converters, according to [31], without any model prior knowledge of the plan. Another wise way is to combine the advantages of the sliding mode control adaptively and ESO in [32,33] for optoelectronic systems and permanent magnet synchronous motors, respectively.

Motivated by the challenges above, this study proposes the AESO to balance the tracking performance and disturbance suppression. When there is a disturbance in the system or a change in the operation scenario, the observer bandwidth of the AESO will automatically increase to improve the tracking performance. On the other hand, when the system is in a steady state, the observer bandwidth will automatically decrease to improve the disturbance suppression. The primary contributions of this study are listed as follows:

- Compared to the existing model-based method for the DAB converter, the proposed AESO method can reduce the number of current sensors, thus significantly reducing the system cost;
- The proposed AESO method effectively balances the tracking performance and disturbance suppression compared to a fixed-bandwidth ESO. Consequently, the AESO streamlines the parameter design process for the controller.

The rest of this article is organized as follows: Section 2 introduces the ESO with a fixed observer bandwidth for the DAB converter. Section 3 describes the principle of operation of the proposed AESO. Section 4 implements the proposed AESO through simulations and experiments to verify its effectiveness. Moreover, the proposed AESO is compared with other methods in various operation scenarios. Finally, Section 5 concludes this study and shows future works for the subsequent studies.

## 2. ESO with a Fixed Observer Bandwidth for DAB Converters

**Figure 1:**
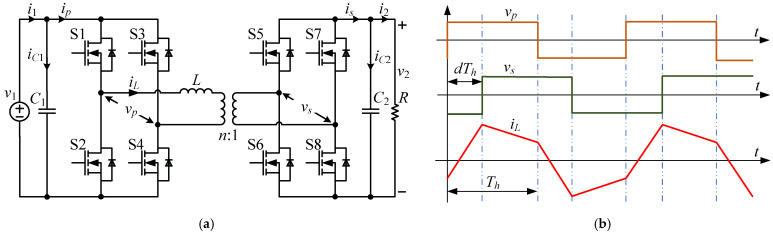
DAB converter: (**a**) topology; and (**b**) waveforms.

Figure 1a shows the typical DAB converter. Two active bridges are connected through an inductor L and a transformer. The capacitors $C_{1}$ and $C_{2}$ on the side of the converter are responsible for the input and output, respectively. The waveforms of the DAB converter are shown in Figure 1b. d represents the phase-shift ratio between the primary and secondary bridges. Every switch operates under the switching frequency f and $T_{h}=T/2=1/\left(2f\right)$ represents the half-switching period.

According to [3], it has been proven that the reduced-order model is more effective than other models in terms of complexity and precision. Thus, the reduced-order model will be applied in this study to illustrate the proposed idea thoroughly. As a result, the secondary side current of the DAB ($i_{s}$) can be determined in the following manner:

(1) $$i_{s}=\frac{nv_{1}}{2fL}d\left(1-d\right)$$

When the value of d is in the range of [0~1], power is transmitted from the left bridge to the right bridge with a maximum value at d = 0.5. However, to simplify the analysis, the phase-shift ratio d is 0≤d≤0.5. Based on (1), the dynamic equation of the output voltage ($v_{2}$) is as follows:

(2) $${\dot{v}}_{2}=\frac{i_{s}-i_{2}}{C_{2}}=\frac{nv_{1}}{2fLC_{2}}d\left(1-d\right)-\frac{i_{2}}{C_{2}}.$$

Rewriting (2), we obtain

(3) $${\dot{v}}_{2}=\alpha u_{d}+F$$

where

(4) $$\alpha =\frac{nv_{1}}{2fLC_{2}}$$

(5) $$u_{d}=d\left(1-d\right)$$

(6) $$F=-\frac{i_{2}}{C_{2}}.$$

Rearranging (3) into the form of state-space, we obtain

(7) $$\left\{\begin{array}{l} {\dot{z}}_{1}=z_{2}+\alpha u_{d}\\ {\dot{z}}_{2}=h \end{array}\right.$$

where

(8) $$\left\{\begin{array}{l} z_{1}=v_{2}\\ z_{2}=F. \end{array}\right.$$

In (7), h is the derivative of the lumped disturbance, which is bounded [34].

From (7), the ESO with a fixed observer bandwidth is constructed where $v_{2}$ and F are regarded as the state variables, as follows:

(9) $$\left\{\begin{array}{l} e_{v}=z_{1}-{\hat{z}}_{1}\\ {\dot{\hat{z}}}_{1}={\hat{z}}_{2}+\alpha u_{d}+\beta_{1}e_{v}\\ {\dot{\hat{z}}}_{2}=\beta_{2}e_{v}. \end{array}\right.$$

In (9), $e_{v}$ is the state observer error, and $\beta_{1}=2\omega ,$ $\beta_{2}=\omega^{2}$, and ω are the observer bandwidth [35,36,37]. Meanwhile, the characteristic equation of the ESO with a fixed observer bandwidth can be deduced as follows:

(10) $$\lambda \left(s\right)={\left(s+\omega \right)}^{2}.$$

On the other hand, to implement the system model dynamically into the digital signal processor (DSP) efficiently, the first-order forward approximation can be utilized because it is one of the most straightforward approximation methods. Thus, the first-order forward approximation is used to discretize (3) at the (*k* + 1)th sampling cycle and $v_{2}$ is set to the reference value ($v_{2ref}$); the value of $u_{d}$ can be calculated as follows [11]:

(11) $$u_{d}\left[k\right]=\frac{v_{2}\left[k+1\right]-v_{2}\left[k\right]}{T\alpha \left[k\right]}-\frac{\hat{F}\left[k\right]}{\alpha \left[k\right]}=\frac{v_{2ref}-v_{2}\left[k\right]}{T\alpha \left[k\right]}-\frac{\hat{F}\left[k\right]}{\alpha \left[k\right]}.$$

Accordingly, the phase-shift ratio d at the *k*th sampling cycle can be directly obtained as follows:

(12) $$d\left[k\right]=\frac{1}{2}-\sqrt{\frac{1}{4}-u_{d}\left[k\right]}.$$

**Figure 2:**
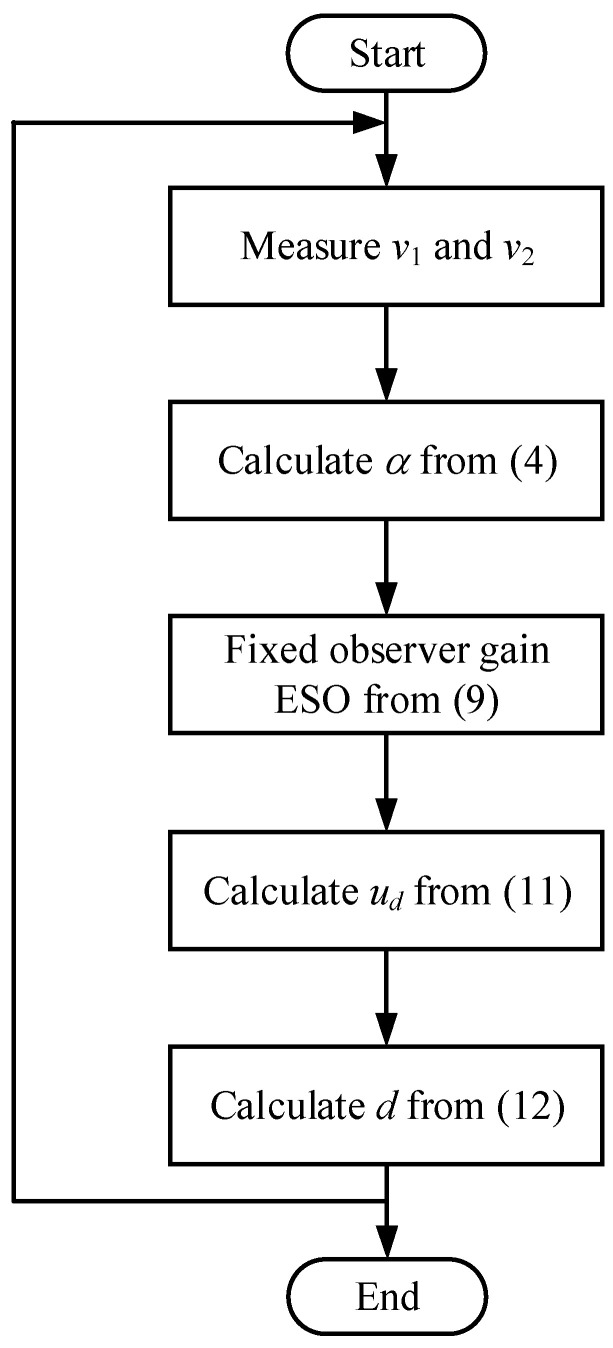
Flowchart of the conventional ESO.

Based on this, the ESO with a fixed observer bandwidth can be implemented, as shown in Figure 2. First, the input and output voltages are measured. After that, α is calculated from (4). The ESO with a fixed observer bandwidth is calculated according to (9), resulting in the observer value $\hat{F}$ can be obtained. Subsequently, $u_{d}$ and d can be obtained from (11) and (12), respectively. Finally, a pulse width modulation is implemented through a gate driver to control the converter.

Obviously, according to (9)–(10), the observer bandwidth ω of ESO must be suitably selected to achieve system stability and robustness. As a result, the dynamic performance is limited because the converter will operate in various scenarios with various disturbances and noises. Therefore, in order to analyze and discuss the impact of the observer bandwidth accurately, the transfer functions in the Laplace transform are derived from (9), as follows:

(13) $${\hat{z}}_{1}\left(s\right)=v_{2}\left(s\right)\frac{\beta_{1}s+\beta_{2}}{s^{2}+\beta_{1}s+\beta_{2}}+x\left(s\right)\frac{s}{s^{2}+\beta_{1}s+\beta_{2}}$$

(14) $${\hat{z}}_{2}\left(s\right)=v_{2}\left(s\right)\frac{\beta_{2}s}{s^{2}+\beta_{1}s+\beta_{2}}-x\left(s\right)\frac{\beta_{2}}{s^{2}+\beta_{1}s+\beta_{2}}$$

where $x=\alpha u_{d}$.

From (13) and (14), the following transfer functions can be obtained:

(15) $$H_{1}\left(s\right)={\left.\frac{{\hat{z}}_{1}\left(s\right)}{v_{2}\left(s\right)}\right|}_{x\left(s\right)=0}=\frac{\beta_{1}s+\beta_{2}}{s^{2}+\beta_{1}s+\beta_{2}}$$

(16) $$H_{2}\left(s\right)={\left.\frac{{\hat{z}}_{2}\left(s\right)}{v_{2}\left(s\right)}\right|}_{x\left(s\right)=0}=\frac{\beta_{2}s}{s^{2}+\beta_{1}s+\beta_{2}}$$

(17) $$H_{3}\left(s\right)={\left.\frac{{\hat{z}}_{1}\left(s\right)}{x\left(s\right)}\right|}_{v_{2}\left(s\right)=0}=\frac{s}{s^{2}+\beta_{1}s+\beta_{2}}$$

(18) $$H_{4}\left(s\right)={\left.\frac{{\hat{z}}_{2}\left(s\right)}{x\left(s\right)}\right|}_{v_{2}\left(s\right)=0}=-\frac{\beta_{2}}{s^{2}+\beta_{1}s+\beta_{2}}$$

where $H_{1}\left(s\right)$ and $H_{2}\left(s\right)$ are the transfer functions of the observer states $z_{1}\left(s\right)$ and $z_{2}\left(s\right)$ against measurement noise, respectively. On the other hand, $H_{3}\left(s\right)$ and $H_{4}\left(s\right)$ are the transfer functions of the observer states $z_{1}\left(s\right)$ and $z_{2}\left(s\right)$ against total disturbances, which consider internal and external model uncertainties, respectively.

## 3. Proposed AESO

In order to overcome the limitation of the ESO with a fixed observer bandwidth, as analyzed and discussed in Section 2, this section presents a detailed analysis of the AESO, which shows how the observer bandwidth can be adjusted automatically to improve the performance of the AESO in the transient progress and steady state. Moreover, the stability of the proposed method is analyzed.

### 3.1. Principle of AESO

Based on the ESO with a fixed observer bandwidth, the AESO is configured as follows:

(19) $$\left\{\begin{array}{l} {\dot{\hat{z}}}_{1}={\hat{z}}_{2}+\alpha u_{d}+\beta_{1A}e_{v}\\ {\dot{\hat{z}}}_{2}=\beta_{2A}e_{v} \end{array}\right.$$

where $\beta_{1A}=2\omega_{A}$, $\beta_{2A}=2\omega_{A}^{2}$, and $\omega_{A}$ is the observer bandwidth of the AESO, expressed in (20):

(20) $$\omega_{A}=\omega_{A,\min}+\left(\omega_{A,\max}-\omega_{A,\min}\right)\frac{2}{\pi }\mathrm{atan}\left(\gamma \left|e_{v}\right|\right)$$

where γ is the positive coefficient. The observer bandwidth $\omega_{A}$ is adjusted between their limitations $\omega_{A,\min}$ and $\omega_{A,\max}$.

**Figure 3:**
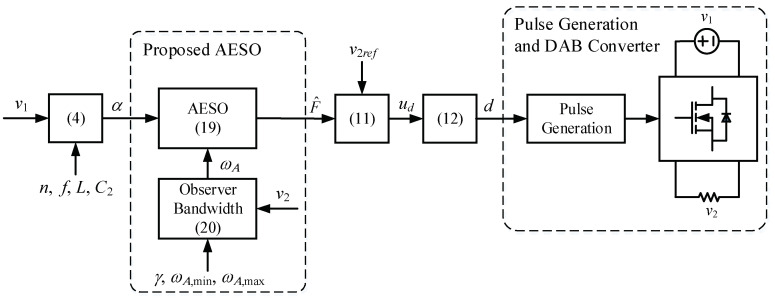
Block diagram of the proposed AESO.

**Figure 4:**
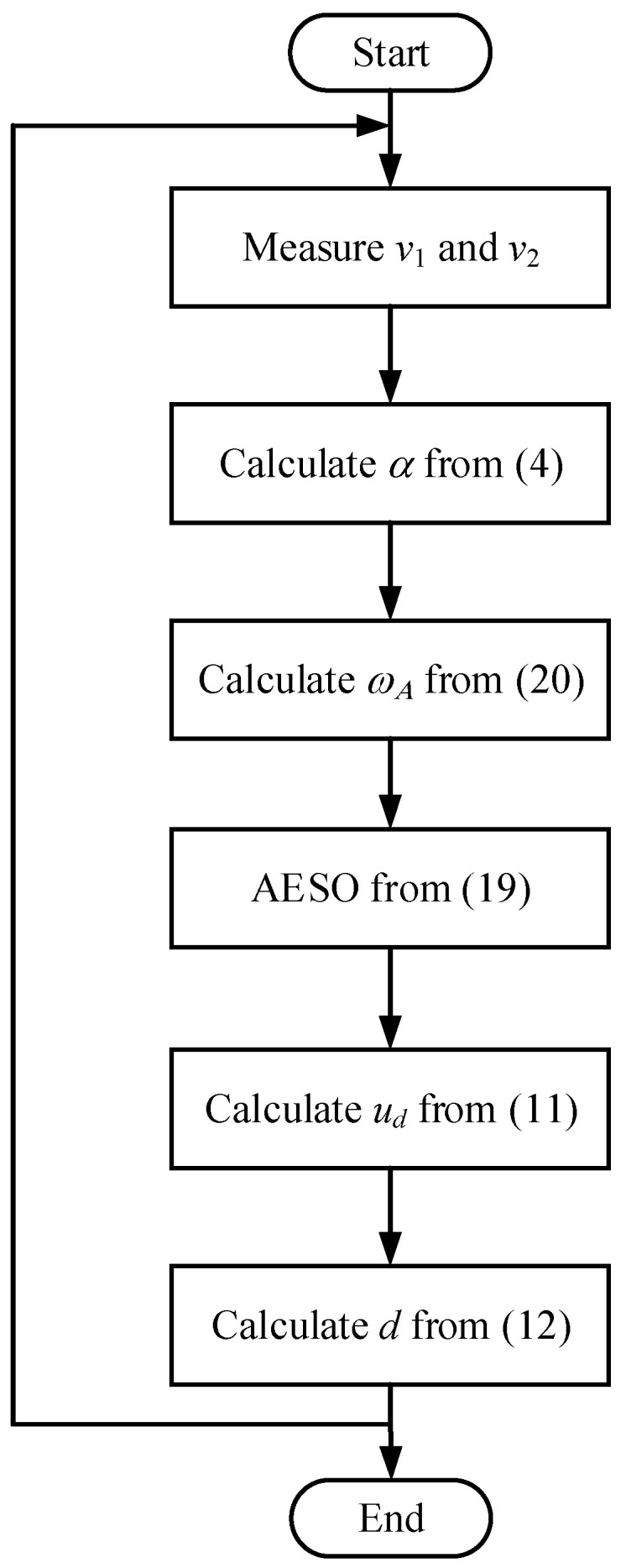
Flowchart of the proposed AESO.

Figure 3 and Figure 4 show the block diagram and flowchart of the proposed AESO for the DAB converter, respectively. First, the input and output voltages are measured. Then, α is calculated from (4). After that, the adaptive observer bandwidth $\omega_{A}$ is calculated according to (20), followed by the AESO obtained from (19). Then, the observer state $\hat{F}$ can be obtained. Subsequently, $u_{d}$ and d are obtained from (11) and (12), respectively. Finally, a pulse width modulation is implemented through a gate driver to control the converter.

### 3.2. Stability Analysis

When assessing the quality of a controller, the stability index is a crucial aspect to consider. Thus, in order to analyze the stability of the proposed AESO, the error equation of the AESO is deduced as follows:

(21) $$\dot{e}=Ae+Bh$$

where

(22) $$\begin{array}{ccc} e={\left[\begin{array}{cc} e_{v} & e_{f} \end{array}\right]}^{T}, & A=\left[\begin{array}{cc} -\beta_{1A} & 0\\ -\beta_{2A} & 0 \end{array}\right], & B={\left[\begin{array}{cc} 0 & 1 \end{array}\right]}^{T}. \end{array}$$

In (22), $e_{f}$ is the disturbance observer error, which is calculated as $e_{f}=z_{2}-{\hat{z}}_{2}$.

Obviously, matrix A is the Hurwitz matrix because $\beta_{1A}$ and $\beta_{2A}$ are bounded. On the other hand, the derivative of the lumped disturbance h is assumed to be bounded. Therefore, the proposed AESO is asymptotically stable, according to [38]. This results in an outcome where, whenever $\hat{F}$ is obtained, the output voltage will track its references, as shown in (11)–(12). Therefore, it is clear that the closed-loop system is asymptotically stable.

## 4. Simulation and Experiment Verification

### 4.1. Simulation

**Figure 5:**
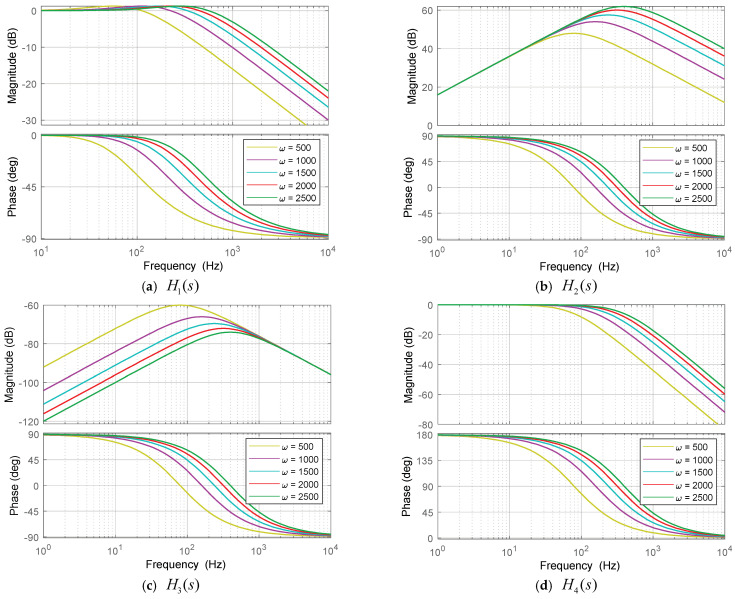
Bode diagrams for the transfer functions of the observer state with various observer bandwidths.

This section compares the proposed AESO to the ESOs with a fixed observer bandwidth, including the low observer bandwidth (LESO) and the high observer bandwidth (HESO). Observer bandwidths of the LESO and the HESO are chosen the same as $\omega_{A,\min}$ and $\omega_{A,\max}$, respectively. On the other hand, the improved model-based phase-shift control (MPSC) performed better than the conventional model-based control, according to [22]. Thus, the proposed AESO is only compared to the MPSC. The detailed expression and control parameters of the MPSC are designed and presented in the Appendix A. Moreover, to choose the control parameters of the AESO, the Bode diagrams for the transfer functions of the observer state with various observer bandwidths are shown in Figure 5 according to (15)–(18). The voltage tracking performance is better if the observer operates with higher bandwidths, as shown in Figure 5a,b. However, a higher observer bandwidth also results in amplifying the higher measurement noise. On the other hand, as shown in Figure 5c,d, poor disturbance suppression with a high bandwidth is present. In other words, the disturbance suppression performance gradually weakens as the observer bandwidth increases. Therefore, it is clear that there is a tradeoff between tracking performance and disturbance suppression [34].

**Figure 6:**
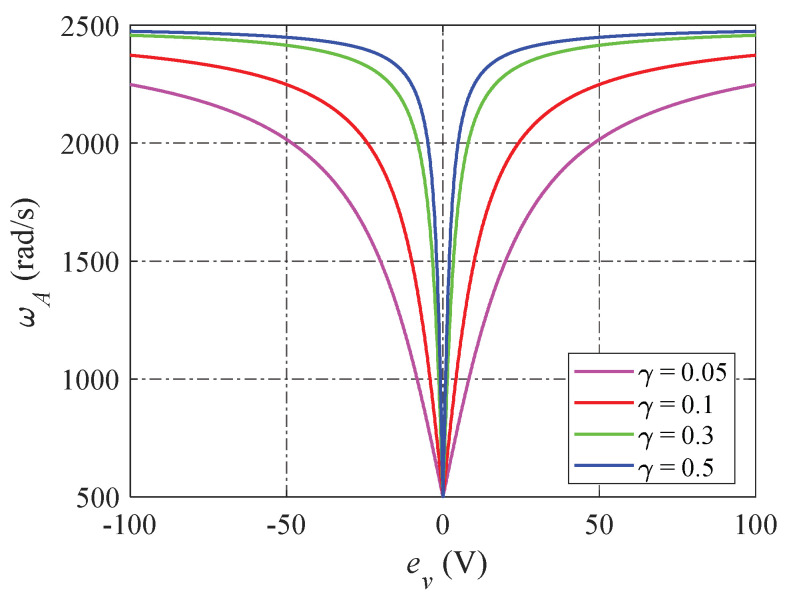
Function relationships of $\omega_{A}$ according to $e_{v}$.

According to (20), the function relationships of $\omega_{A}$ according to the voltage error $e_{v}$ under various values of γ are shown in Figure 6. Obviously, $e_{v}$ is decreased faster with a higher observer bandwidth. That means, when the transient process is significant, the bandwidth of the AESO is high, ensuring system convergence occurs as quickly as possible. On the other hand, in the steady state, the disturbance sensitivity is significantly decreased because the bandwidth of the AESO decreases. Moreover, $\omega_{A}$ has a smooth change within observer bandwidths $\omega_{A,\min}$ and $\omega_{A,\max}$, resulting in the AESO achieving the best performance in the tradeoff of tracking performance and disturbance suppression.

From the analysis above, the parameters of the DAB converter and the proposed AESO in the simulation are shown in Table 1 and Table 2, respectively.

**Figure 7:**
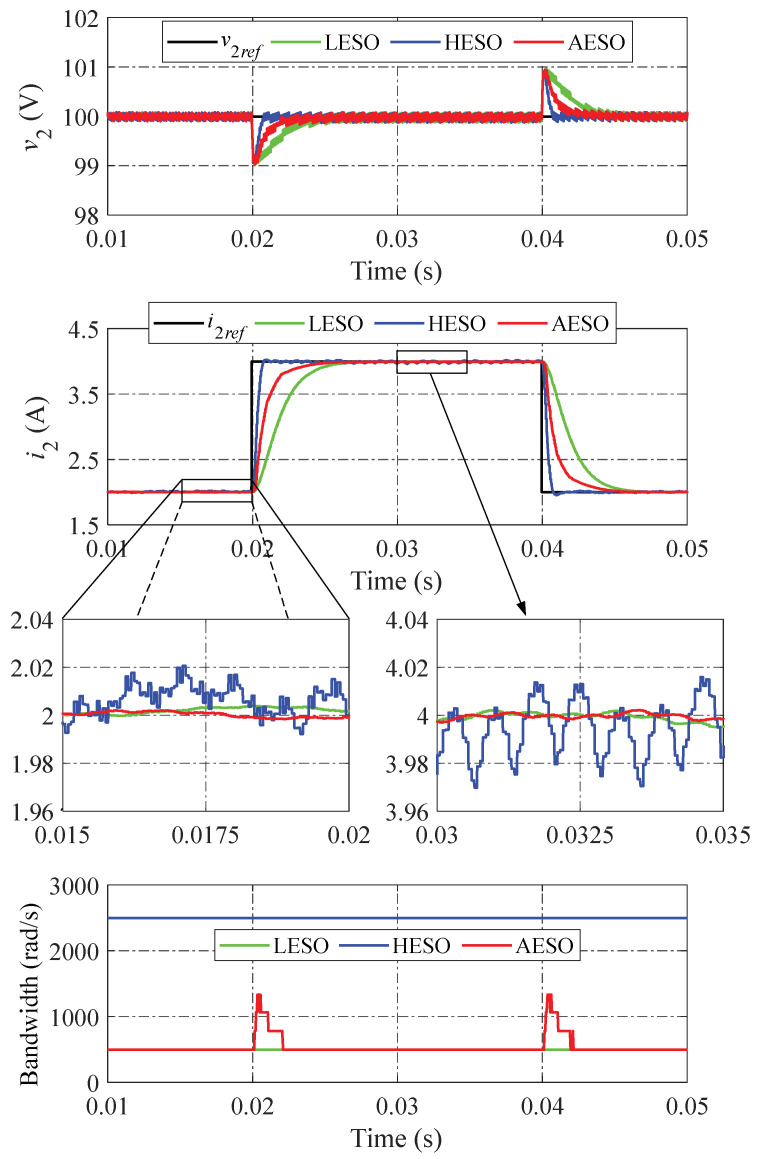
Simulation results when changing the load current.

The simulation results of the step load change performed by the LESO, the HESO, and the proposed AESO are shown in Figure 7. All three observer methods exhibit the identical undershoot and overshoot of $v_{2}$ when the output current $i_{2}$ suddenly steps up and down at 0.02 s and 0.04 s, respectively. Compared to the proposed AESO and LESO, the voltage tracking performance of the HESO is somewhat faster. However, the observed current of the HESO fluctuates more strongly than the proposed method’s, meaning the observer performance of the HESO is lower than that of other controllers. The reason for this is that the proposed AESO can promptly increase the observer bandwidth in the event of a sudden change in $i_{2}$, resulting in reducing the voltage error as soon as possible while simultaneously assuring the suppression of the total disturbances. When the voltage $v_{2}$ tracks to its reference $v_{2ref}$ in the steady state, the bandwidth of the proposed AESO automatically decreases to $\omega_{A,\min}$. This causes the observed current to track the actual current smoothly. On the other hand, compared to the other controllers, the LESO has the worst dynamic performance, with the longest settling time of the output voltage and the longest settling time of the observed current. However, the observed current in the LESO provides a slight fluctuation. It also fails to achieve a fast convergence due to the small value of the observer bandwidth. In comparison to the LESO and HESO, it is evident that the proposed AESO demonstrates the best tracking performance and disturbance suppression when the output current $i_{2}$ suddenly changes. Moreover, it is easy to see that the observed load currents in all methods are perfectly consistent with the reference value ($i_{2ref}$). This is because of the ability to reject the load disturbance and the robustness against the uncertainties of the ESO, which is regularly utilized in high-performance applications, especially power converter control systems.

**Figure 8:**
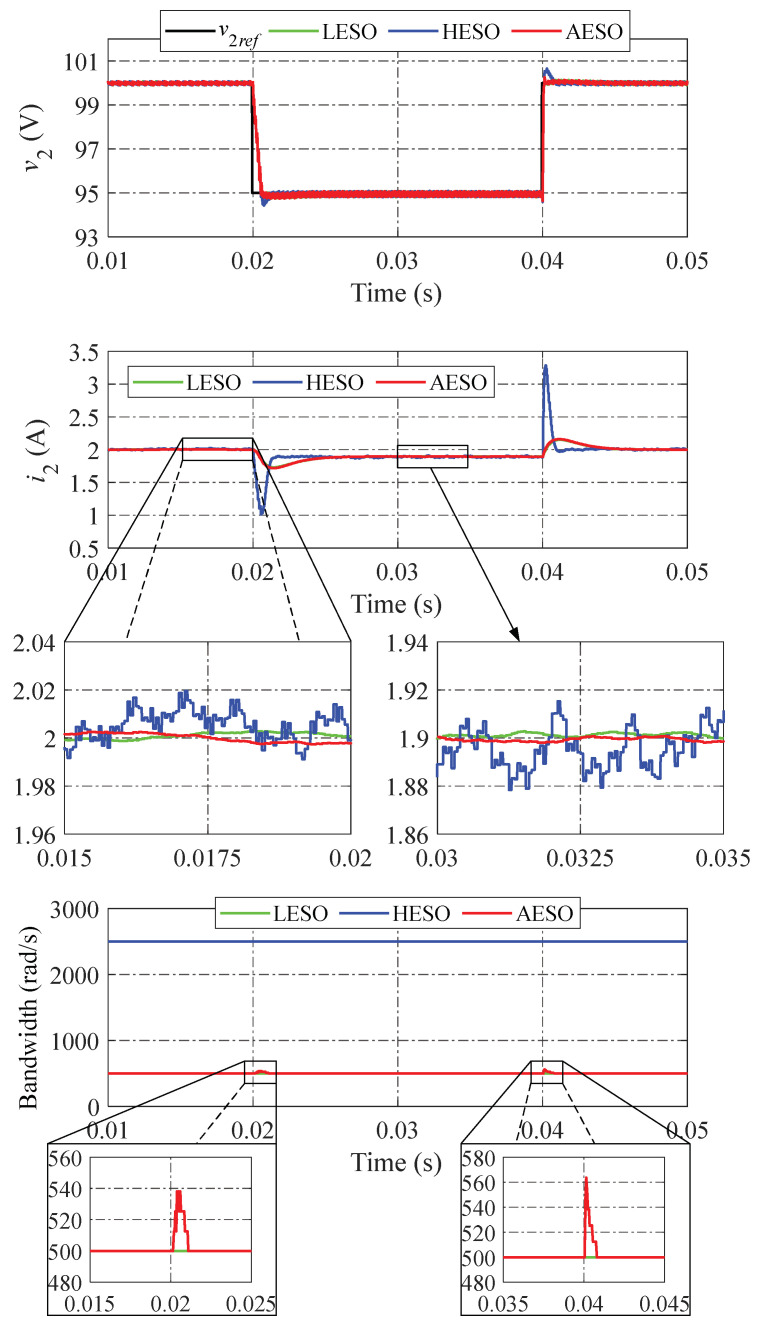
Simulation results when changing the voltage reference.

The simulation of the LESO, the HESO, and the proposed AESO is shown in Figure 8 when the voltage reference $v_{2ref}$ steps down and up between 100 V and 95 V. Obviously, the undershoot and overshoot of $v_{2}$ are both occurrences that can be found in the HESO. while the LESO and the proposed AESO offer a more stable dynamic performance. In this case, the dynamic and observer performances shown by the LESO and the proposed AESO are similar.

**Figure 9:**
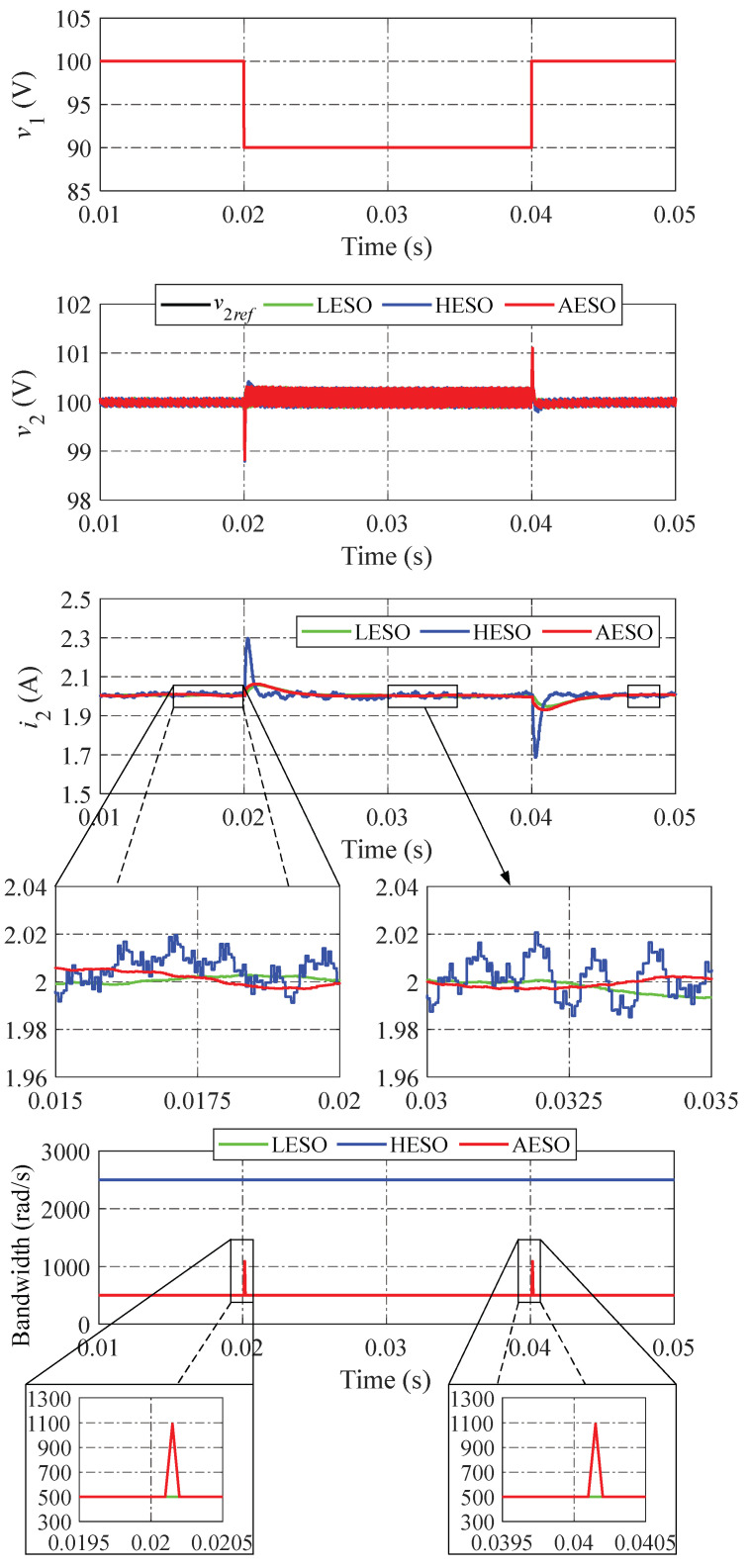
Simulation results when changing the input voltage.

Figure 9 shows the simulation of the LESO, the HESO, and the proposed AESO when changing the input voltage between 100 V and 90 V. Even though all three methods have a dynamic performance comparable to one another, the HESO exhibits a significant fluctuation of the observed current, showing the same phenomenon as in Figure 7 and Figure 8.

The results of the simulations shown in Figure 7, Figure 8 and Figure 9 make it abundantly clear that the observer bandwidth is adaptively adjusted between the minimum and maximum values according to the voltage error, resulting in an improved dynamic performance under various operation scenarios and a better disturbance suppression in the steady state. In other words, the proposed AESO has a superior tracking performance and disturbance suppression overall compared to both the LESO and HESO.

**Figure 10:**
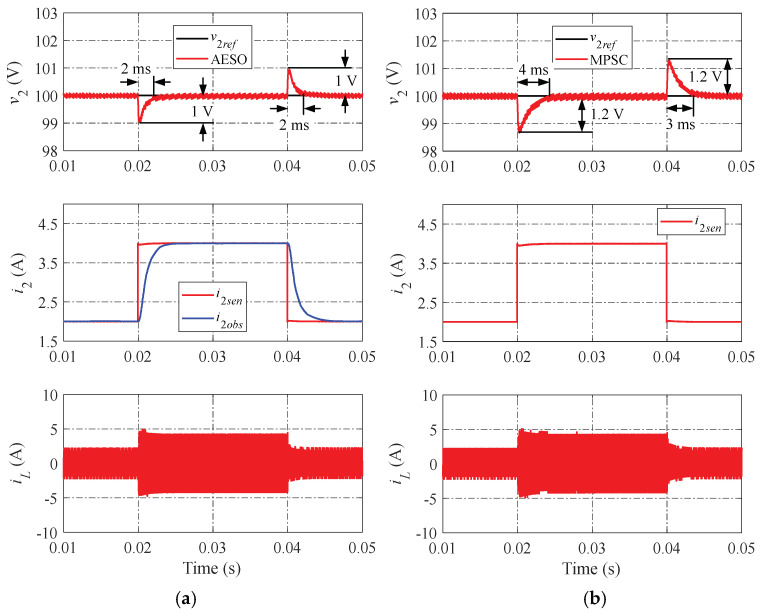
Simulation results when changing the load current: (**a**) proposed AESO; and (**b**) MPSC.

**Figure 11:**
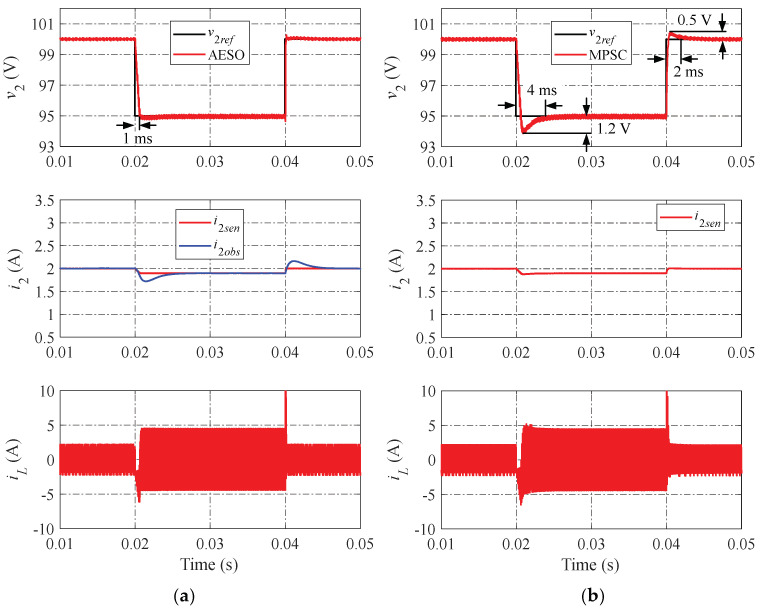
Simulation results when changing the voltage reference: (**a**) proposed AESO; and (**b**) MPSC.

**Figure 12:**
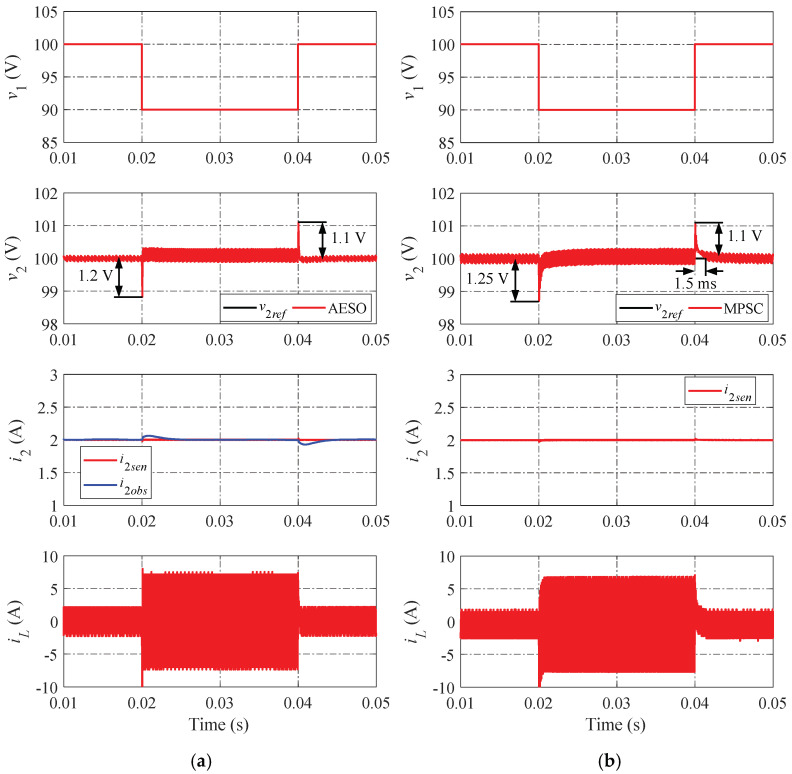
Simulation results when changing the input voltage: (**a**) proposed AESO; and (**b**) MPSC.

The simulation results of the proposed AESO and the MPSC are shown in Figure 10, Figure 11 and Figure 12 in various operation scenarios of the DAB converter for a further comparison of the dynamic performance. Figure 10 shows the simulation results when changing the load current. When the load current changes, the proposed AESO shows a lower peak value of undershoot and overshoot. Moreover, the settling times in the transient progress of the proposed AESO are shorter than that in the MPSC. It can be seen that the proposed AESO demonstrates superior dynamic performance compared to the MPSC. In addition, the observed current ($i_{2obs}$) is totally consistent with the value measured from the sensor ($i_{2sen}$) in the proposed AESO. Figure 11 and Figure 12 demonstrate simulation results similar to those in Figure 10 when changing the voltage reference and the input voltage, respectively.

To further validate the robustness of the proposed AESO against the strong influence of the mismatched system parameters, the simulation comparison should be expanded to include the scenarios in which the parameters are mismatched. Notable with regard to that, the simulations presented above indicate that the effects of changing the voltage reference and the input voltage situations are comparable to those of changing the load current. Consequently, the case of changing the load current is utilized as a typical representation for comparison in the following simulations.

On the other hand, the inductor is no longer incorporated following the AESO principle, which is demonstrated in (19). Additionally, the proposed AESO that has been presented can compensate for the voltage error that occurs in the steady state. In addition, according to [39], the ESO and the parameter identification technique use the output voltage error. This leads to a conflict and a decline in performance if the ESO and the parameter identification technique are utilized simultaneously. The consequence is that this study does not consider the effect of the inductor mismatch. According to [40], the value of the capacitor may experience slight variations over time due to temperature drift, manufacturing tolerance, age, and operating circumstances. Consequently, a variation of 20% of parameter mismatches may occasionally be detected [41,42]. Therefore, the simulation results that occur when $C_{2}$ varies by ±20% are utilized to demonstrate the comparability.

**Figure 13:**
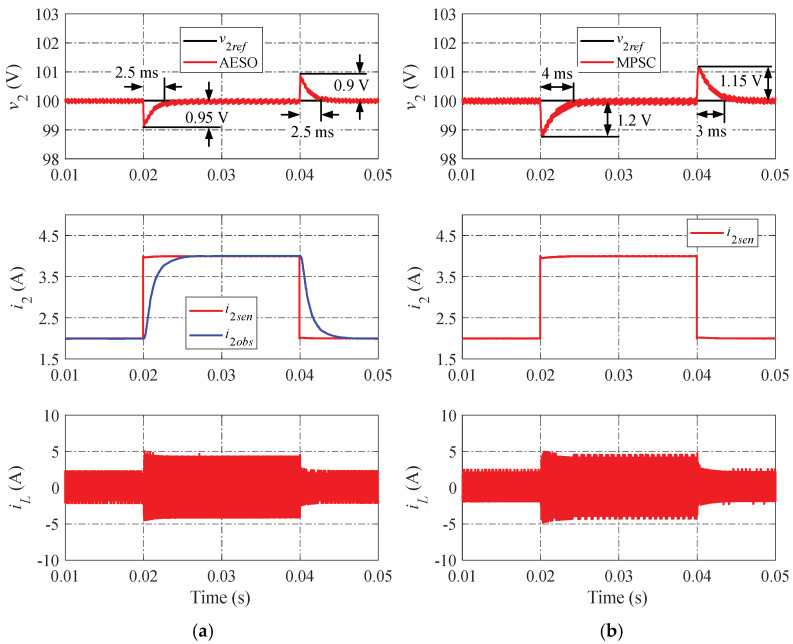
Simulation results when changing the load current under a mismatch case of $C_{2}$ with +20%: (**a**) proposed AESO; and (**b**) MPSC.

**Figure 14:**
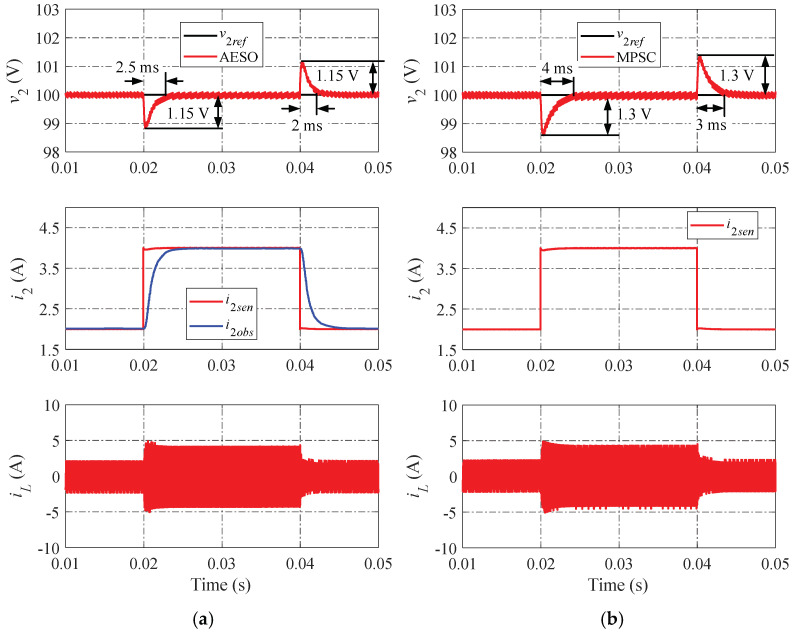
Simulation results when changing the load current under a mismatch case of $C_{2}$ with −20%: (**a**) proposed AESO; and (**b**) MPSC.

Figure 13 and Figure 14 show the simulation results when changing the load current under the mismatch cases of $C_{2}$ with +20% and −20% of the proposed AESO and the MPSC, respectively. Obviously, these results confirm that the proposed AESO performs better than the MPSC in mismatch cases.

### 4.2. Experiment

**Figure 15:**
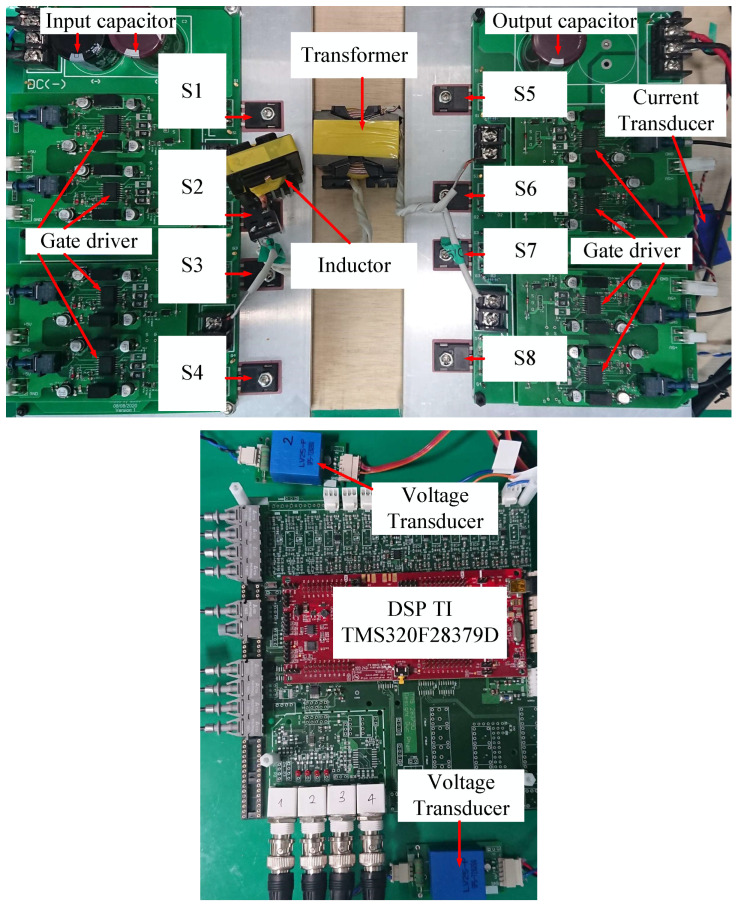
Hardware prototype.

A hardware prototype is built to validate the effectiveness of the proposed AESO experimentally, as shown in Figure 15. The hardware components of the DAB converter are shown in Table 3. All actual values of the hardware components are measured by electronic equipment in the laboratory to identify their actual values, as shown in Table 4. The parameters of the proposed AESO in the experiment are shown in Table 2. In this section, the performance verification of the proposed AESO is compared to the MPSC experimentally when the load current is changed. Moreover, the cases of mismatched capacitors are also investigated.

**Figure 16:**
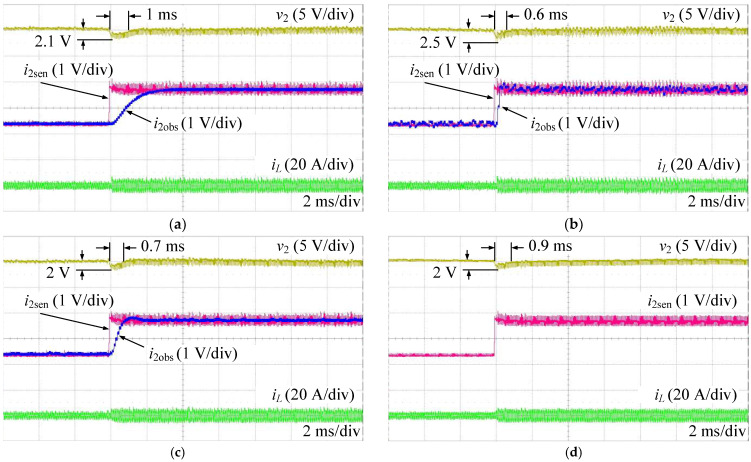
Experimental results when increasing the load current. (**a**) LESO; (**b**) HESO; (**c**) AESO; (**d**) MPSC.

Figure 16 shows the experimental results when increasing the load current from 1.4 A to 2.8 A. The observed currents ($i_{2obs}$) are totally consistent with the values measured from the sensor ($i_{2sen}$) in all ESO methods, as shown in Figure 16a–c. Compared to the proposed AESO, the performance of the HESO in terms of voltage tracking somewhat increases with a shorter settling time. On the other hand, the observed current of the HESO, as shown in Figure 16b, exhibits a greater degree of fluctuation than those in the LESO and the proposed AESO, as shown in Figure 16a,c. Conversely, the LESO shows a worse dynamic performance than the HESO and the proposed ESO, where it has the longest output voltage settling time. However, the LESO has a very smooth observer current. That confirms the tracking performance and disturbance suppression tradeoff, as discussed above. Obviously, the proposed AESO shows the best tracking performance and disturbance suppression compared to the LESO and the HESO. Moreover, as shown in Figure 16c,d, compared to the MPSC, it is not difficult to notice that the proposed AESO demonstrates a better dynamic performance with a shorter settling time when the load current increases.

**Figure 17:**
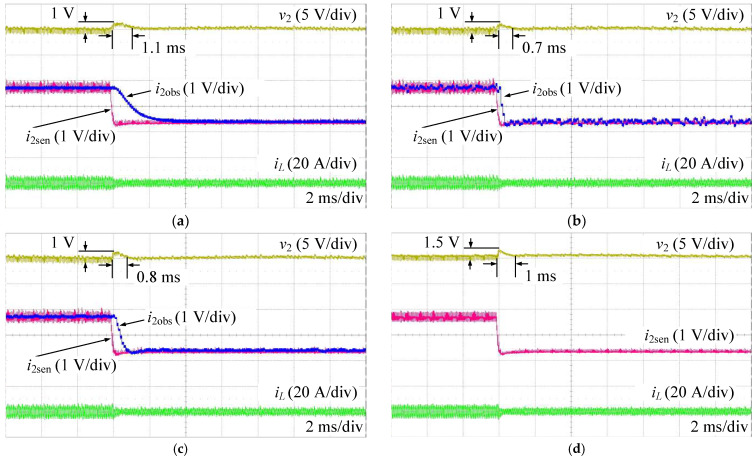
Experimental results when decreasing the load current: (**a**) LESO; (**b**) HESO; (**c**) AESO; and (**d**) MPSC.

On the other hand, Figure 17 shows the experimental results when decreasing the load current from 2.8 A to 1.4 A. This scenario also shows similar performances, as shown in Figure 16.

**Figure 18:**
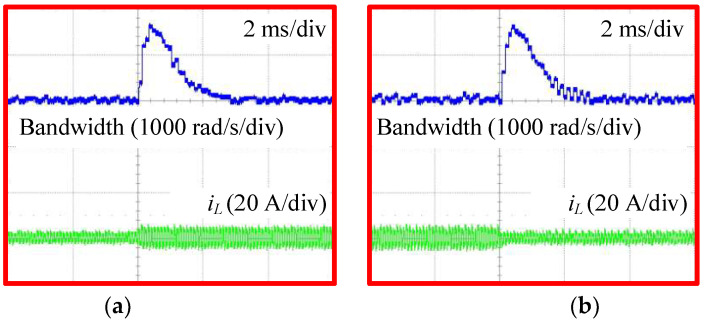
Variations of the bandwidth in the AESO: (**a**) increasing the load current; and (**b**) decreasing the load current.

Figure 18 shows the variations in the bandwidth of the proposed AESO when increasing and decreasing the load current, detected under simultaneous conditions as in Figure 16c and Figure 17c, respectively. At the moment the load current changes, the bandwidth of the proposed AESO automatically increases from the minimum value $\omega_{A,\min}$ in order to compensate for the change in voltage error. After that, it decreases again to $\omega_{A,\min}$ in the steady state. The peak value of the bandwidth in these cases is around 1600 rad/s. That confirms the effectiveness of the proposed AESO with the same performance as shown in the simulation.

**Figure 19:**
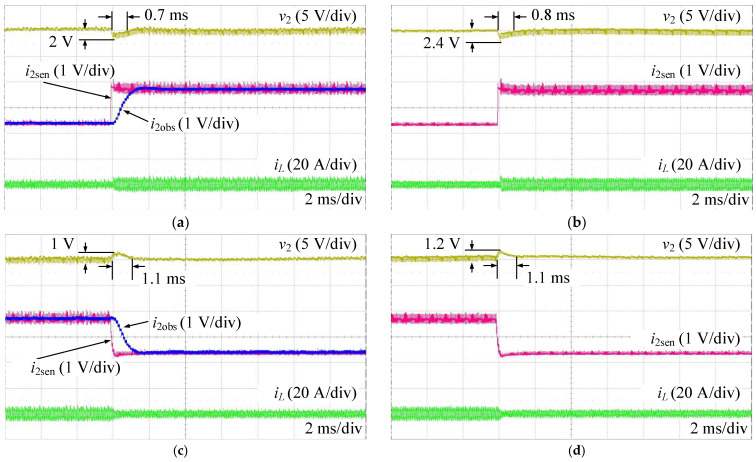
Experimental results when changing the load current under a mismatch case of $C_{2}$ with +20%: (**a**) AESO when increasing the load current; (**b**) MPSC when increasing the load current; (**c**) AESO when decreasing the load current; and (**d**) MPSC when decreasing the load current.

Figure 19 shows the experimental results of the proposed AESO and the MPSC when changing the load current under a mismatch case of $C_{2}$ with +20%. It is easy to see that the dynamic performances of $v_{2}$ in the proposed AESO are better than that of the MPSC. Thus, the proposed AESO also shows better dynamics than the MPSC. In addition, the observed load currents of the proposed AESO, in this case, are also perfectly consistent with the values measured by the sensor.

**Figure 20:**
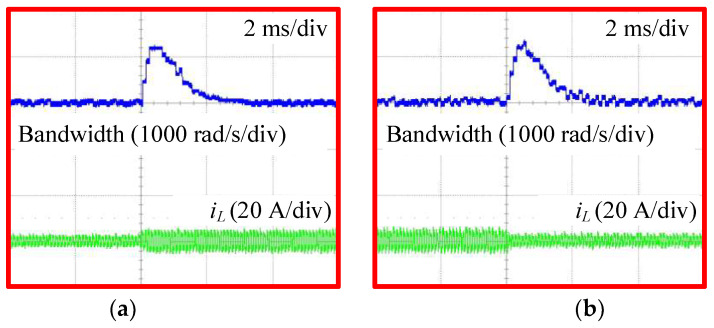
Variations of the bandwidth in the AESO under a mismatch case of $C_{2}$ with +20%: (**a**) increasing the load current; and (**b**) decreasing the load current.

Figure 20 shows the variations in the bandwidth of the proposed AESO under simultaneous conditions, as shown in Figure 19. When the load current changes, the bandwidth of the proposed AESO automatically increases, and then decreases.

**Figure 21:**
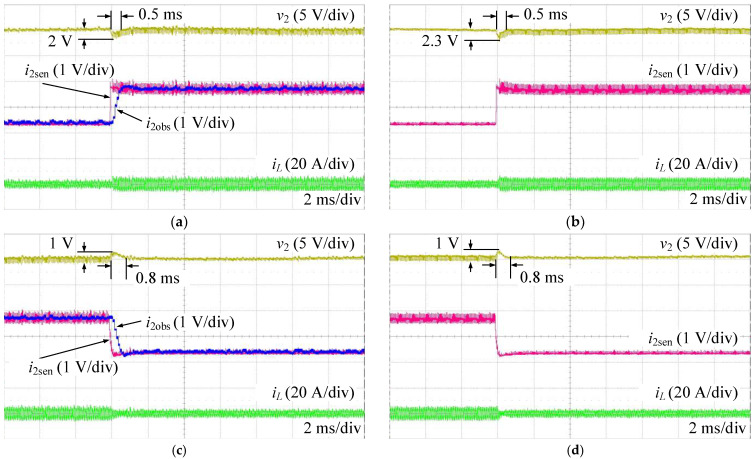
Experimental results when changing the load current under a mismatch case of $C_{2}$ with −20%: (**a**) AESO when increasing the load current; (**b**) MPSC when increasing the load current; (**c**) AESO when decreasing the load current; and (**d**) MPSC when decreasing the load current.

**Figure 22:**
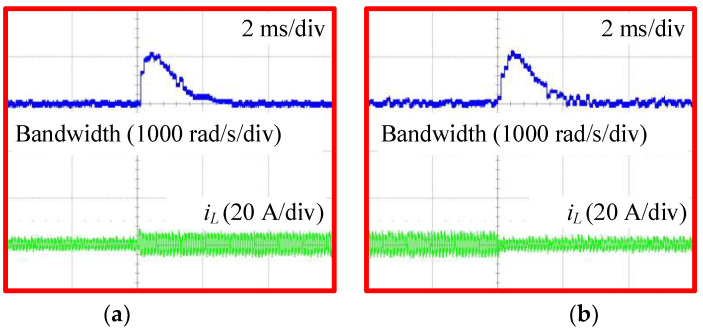
Variations of the bandwidth in the AESO under a mismatch case of $C_{2}$ with −20%: (**a**) increasing the load current; and (**b**) decreasing the load current.

Similarly, Figure 21 shows the experimental results of the proposed AESO and the MPSC when changing the load current under a mismatch case of $C_{2}$ with −20%. Figure 22 shows the variations in the bandwidth of the proposed AESO under simultaneous conditions, as shown in Figure 21. In this case, both methods show similar dynamic performances. In addition, the observed load currents of the proposed AESO are also perfectly consistent with the values measured by the sensor.

Moreover, for synthesizing the overall performances of the proposed AESO compared to the others, Table 5 briefly compares the significant peak values that demonstrated the dynamic performance of the LESO, HESO, AESO, and MPSC.

Finally, Table 6 shows overall comparisons of the proposed AESO method and the MPSC method.

## 5. Conclusions

This study proposed the AESO for the DAB converters. Effectively compromising the tracking performance and disturbance suppression, the proposed AESO can eliminate the current sensor and significantly increase the dynamic performance compared to the ESO with a fixed observer bandwidth and the MPSC in almost all matched and mismatched capacitor cases. This is accomplished by automatically adjusting the observer bandwidth according to the change in voltage error. Thus, the proposed AESO can balance the tracking performance and disturbance suppression. The proposed AESO’s effectiveness was proven compared to the ESO with a fixed observer bandwidth and the MPSC through simulations and experiments in various operation circumstances.

In the upcoming studies, additional mathematical models of parameter sensitivity will be carried out to demonstrate the proposed strategy’s effectiveness further. In addition, the AESO that has been developed will be combined with other modulation techniques to optimize the control flexibility and converter efficiency.

## Figures and Tables

**Table 1:** Simulation parameters of the DAB converter.

Symbol	Description	Value
$v_{1}$	Input voltage	100 V
$v_{2ref}$	Output voltage reference	100 V
n	Transformer turn ratio	1
f	Switching frequency	10 kHz
L	Series inductance	50 μH
$C_{1}$	Input capacitance	440 μF
$C_{2}$	Output capacitance	220 μF
R	Resistive load	50 Ω

**Table 2:** Parameters of AESO.

Symbol	Description	Value
$\omega_{A,\min}$	Minimum observer bandwidth	500 rad/s
$\omega_{A,\max}$	Maximum observer bandwidth	2500 rad/s
γ	Positive coefficient	0.1

**Table 3:** Hardware components.

Component	Description	Value
Switching devices	C3M0065090D × 8	*V_DS_* = 900 V, *I_D_* = 36 A
Input capacitors	Panasonic UQ Samyoung TDA	450 V, 220 μF 450 V, 220 μF
Output capacitors	Samyoung TDA	450 V, 220 μF
Transformer	TDK PQ50/50, Ferrite Core, Litz wire 0.1 mm × 140 strands	31:31 turns
Inductor	TDK EI40, Ferrite Core, Litz wire 0.1 mm × 140 strands	24 turns
Voltage sensors	LV 25-P	*t* = 40 μs
Current sensors	LA 55-P	BW (–1 dB) 200 kHz

**Table 4:** Experiment parameters of the DAB converter.

Symbol	Description	Value
$v_{1}$	Input voltage	80 V
$v_{2ref}$	Output voltage reference	80 V
n	Transformer turn ratio	1
f	Switching frequency	10 kHz
L	Series inductance	51 μH
$C_{1}$	Input capacitance	431 μF
$C_{2}$	Output capacitance	219 μF
R	Resistive load	57 Ω

**Table 5:** Comparisons of dynamics performance of LESO, HESO, AESO, and MPSC.

Validation	Operation Scenario	Overshoot/Undershoot				Settling Time			
		LESO	HESO	AESO	MPSC	LESO	HESO	AESO	MPSC
Simulation	Changing the load current	1 V	1 V	1 V	1.2 V	4 ms	3 ms	2 ms	4 ms
	Changing the voltage reference	0.2 V	0.4 V	0.2 V	1.2 V	1 ms	1 ms	1 ms	4 ms
	Changing the input voltage	1.2 V	1.2 V	1.2 V	1.25 V	0.1 ms	0.1 ms	0.1 ms	1.5 ms
Experiment	Increasing the load current	2.1 V	2.5 V	2 V	2 V	1 ms	0.6 ms	0.7 ms	0.9 ms
	Decreasing the load current	1 V	1 V	1 V	1.5 V	1.1 ms	0.7 ms	0.8 ms	1 ms

**Table 6:** Overall comparisons.

Tasks	MPSC	AESO
Number of current sensors	2	0
Number of voltage sensors	2	2
Observer performance	-	Good
Dynamic performance	Moderate	Good
Robustness to parameter mismatches	Moderate	Good

## Data Availability

Data are contained within the article.

## Appendix A

The improved model-based phase-shift control (MPSC), according to [22], is shown in the following.

The phase-shift ratio of the MPSC is as follows:

(A1) $$d_{MPSC}=\left\{\begin{array}{cc} \frac{1}{2}-\sqrt{\frac{1}{4}-\frac{i_{ref}}{k^{*}}} & 0\leq i_{ref}\leq \frac{k^{*}}{4}\\ -\frac{1}{2}+\sqrt{\frac{1}{4}+\frac{i_{ref}}{k^{*}}} & -\frac{k^{*}}{4}\leq i_{ref}\leq 0 \end{array}\right.$$

where $k^{*}$ and $i_{ref}$ are calculated from (A2) and (A3), respectively.

(A2) $$k^{*}=\frac{n^{*}v_{1ref}}{2fL^{*}}$$

(A3) $$i_{ref}=i_{2}+i_{c\_ref}.$$

In (A2) and (A3), $n^{*}$ and $L^{*}$ represent the theoretical design values, $v_{1ref}$ denotes the reference values of input voltage, and $i_{c\_ref}$ is generated from the feedback control loop with the proportional–integral (PI) control $G_{PI}\left(s\right)$, which is presented as follows:

(A4) $$G_{PI}\left(s\right)=k_{p}\left(1+\frac{1}{sT_{r}}\right)$$

where the proportional gain $k_{p}$ and the integrator time constant $T_{r}$ are calculated as follows:

(A5) $$k_{p}=C_{2}\omega_{c}$$

(A6) $$T_{r}=\frac{\tan\left(\varphi_{m}+\omega_{c}T_{d}\right)}{\omega_{c}}.$$

In (A5) and (A6), $\omega_{c}$ is the cutoff frequency, $\varphi_{m}$ is the specified phase margin, and $T_{d}$ is the control delay.

The parameters of MPSC are shown in Table A1.

**Table A1:** Parameters of MPSC.

Symbol	Description	Value
$\omega_{c}$	Cutoff frequency	2000π rad/s
$\varphi_{m}$	Phase margin	60 deg
$T_{d}$	Control delay	50 μs
$k_{p}$	Proportional gain	1.376
$T_{r}$	Integrator time constant	0.7488 ms
